# Bacterial Profiling Reveals Novel “*Ca*. Neoehrlichia”, *Ehrlichia*, and *Anaplasma* Species in Australian Human-Biting Ticks

**DOI:** 10.1371/journal.pone.0145449

**Published:** 2015-12-28

**Authors:** Alexander W. Gofton, Stephen Doggett, Andrew Ratchford, Charlotte L. Oskam, Andrea Paparini, Una Ryan, Peter Irwin

**Affiliations:** 1 Vector and Water-borne Pathogen Research Group, School of Veterinary and Life Sciences, Murdoch University, Perth, Western Australia, Australia; 2 Department of Medical Entomology, Pathology West and Institute for Clinical Pathology and Medical Research, Westmead Hospital, Westmead, New South Wales, Australia; 3 Emergency Department, Mona Vale Hospital, New South Wales, Australia; Metabiota, UNITED STATES

## Abstract

In Australia, a conclusive aetiology of Lyme disease-like illness in human patients remains elusive, despite growing numbers of people presenting with symptoms attributed to tick bites. In the present study, we surveyed the microbial communities harboured by human-biting ticks from across Australia to identify bacteria that may contribute to this syndrome. Universal PCR primers were used to amplify the V1-2 hyper-variable region of bacterial 16S rRNA genes in DNA samples from individual *Ixodes holocyclus* (*n* = 279), *Amblyomma triguttatum* (*n* = 167), *Haemaphysalis bancrofti* (*n* = 7), and *H*. *longicornis* (*n* = 7) ticks. The 16S amplicons were sequenced on the Illumina MiSeq platform and analysed in USEARCH, QIIME, and BLAST to assign genus and species-level taxonomies. Nested PCR and Sanger sequencing were used to confirm the NGS data and further analyse novel findings. All 460 ticks were negative for *Borrelia* spp. by both NGS and nested PCR analysis. Two novel “*Candidatus* Neoehrlichia” spp. were identified in 12.9% of *I*. *holocyclus* ticks. A novel *Anaplasma* sp. was identified in 1.8% of *A*. *triguttatum* ticks, and a novel *Ehrlichia* sp. was identified in both *A*. *triguttatum* (1.2%) ticks and a single *I*. *holocyclus* (0.6%) tick. Further phylogenetic analysis of novel “*Ca*. Neoehrlichia”, *Anaplasma* and *Ehrlichia* based on 1,265 bp 16S rRNA gene sequences suggests that these are new species. Determining whether these newly discovered organisms cause disease in humans and animals, like closely related bacteria do abroad, is of public health importance and requires further investigation.

## Introduction

Over the last 30 years in Australia there have been reports of an illness in humans, the onset of which has been putatively associated with parasitism by ticks, most frequently the Australian paralysis tick (*Ixodes holocyclus*) [1]. This undetermined disease usually presents as acute flu-like symptoms including headache, fever, and fatigue that can persist for weeks to months, and may develop into a severe chronic illness that can include, but is not limited to, myalgia, arthralgia, chronic migraine, and a systemic inflammatory syndrome [1, 2]. Similarities between these symptoms and those of Lyme disease have led to the controversial diagnosis by some physicians of Lyme disease in Australian patients [3, 4].

In the northern hemisphere, Lyme disease is caused by the bacteria *Borrelia burgdorferi* sensu lato and is transmitted by several species of *Ixodes* ticks, including *I*. *ricinus* and *I*. *persulcatus* in Europe and Asia, and *I*. *scapularis* and *I*. *pacificus* in North America, none of which occur in Australia [5, 6]. *Borrelia burgdorferi* sensu lato is not considered by many physicians to occur in Australia, and over 20 years of scientific effort has failed to find sufficient evidence of *B*. *burgdorferi* sensu lato in Australian ticks, wildlife, or humans that did not acquire *Borrelia* infection overseas [1, 2, 7]. Consequently, there is significant public concern and medical uncertainty over the diagnosis and treatment of a Lyme disease-like illness in Australia, and there is a need for robust scientific inquiry to clarify the aetiology of this illness.

*Ixodes holocyclus* is the most significant Australian tick species from both a medical and veterinary perspective [8]. It is the tick most commonly found parasitising humans and domestic animals in its enzootic range, which spans coastal areas along almost the entire east coast of Australia and includes many of Australia’s most densely populated regions [9]. Its natural wildlife hosts include a variety of small marsupials such as bandicoots (*Isoodon* spp. and *Perameles* spp.) and possums (*Trichosurus vulpecula* and *Pseudocheirus peregrinus*) [8]. *Ixodes holocyclus* causes life-threatening paralysis in domestic animals through envenomation, and in humans it can cause weakness, paralysis, and dermatological and allergic reactions, including mammalian meat allergies [10]. It is also a vector of the human pathogens *Rickettsia australis* and *R*. *honei*, agents of Queensland tick typhus and Flinders Island spotted fever, respectively [11, 12]. On the west coast of Australia, the most common human-biting tick is the ornate kangaroo tick, *Amblyomma triguttatum* [8], which is a putative host of *Coxiella burnetii*, the aetiological agent of Q fever, and the spotted fever pathogen *R*. *gravesii* [8, 13, 14].

Recently, a survey of bacteria harboured by *I*. *holocyclus* ticks from New South Wales (NSW), Australia, using bacterial 16S rRNA gene (16S) profiling, identified four novel candidate pathogens, including a relapsing fever group *Borrelia* sp., an *Anaplasma* sp., and two novel “*Candidatus* Neoehrlichia” species [7]. Phylogenetic analysis of 300 bp 16S rRNA gene sequences from these bacteria revealed that the *Borrelia* and “*Ca*. Neoehrlichia” were closely related to the known human tick-borne pathogens *B*. *duttonii* and “*Ca*. N. mikurensis”, respectively [7], which share some clinical similarities to those described by patients suffering Lyme disease-like illness in Australia [15, 16]. The novel *Anaplasma* sp. was closely related to the tick-borne pathogen of cattle, *A*. *bovis* [7]. None of these candidate pathogens had been described previously in Australia.

The present study was designed in order to better understand the range and genetic diversity of microorganisms potentially transmitted to humans by ticks in Australia. As previously described [7], next-generation sequencing (NGS) and bioinformatics tools were used to profile bacterial populations within ticks removed from people around Australia. Additionally, species-specific PCR assays, Sanger sequencing, and Bayesian phylogenetic reconstructions were implemented to further analyse and confirm results obtained by NGS.

## Methods

### Ethics statement

This research complies with the *Australian Code for the ResponsibleConduct of Research*, 2007, and was approved by the Murdoch UniversityHuman Research Ethics Committee (Permit No. 2011–005). All tickcollections were opportunistic and were volunteered by people who hadeither removed the ticks from themselves, or had them removed by a medicalprofessional during outpatient treatment. Participants provided writtendocumented consent to participate in this study, and the consent procedurewas approved by the Murdoch University Human Research Ethics Committee(Permit No. 2011–005).

### Tick collection and identification

**Fig 1:**
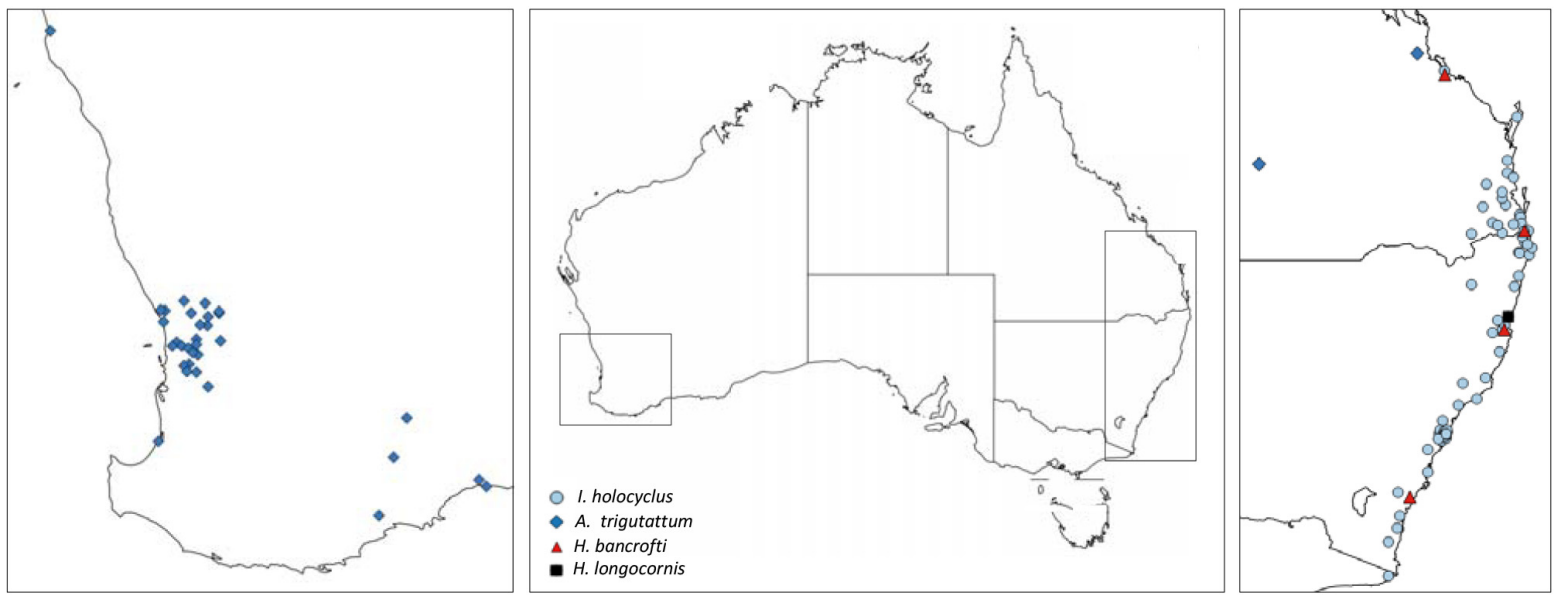
Geographic origin of *I*. *holocyclus*, *A*. *triguttatum*, and *Haemaphysalis* ticks used in this study. Centre, map of Australia; Left, inset of south-west Western Australia; Right, inset of Australian east coast.

A total of 460 individual ticks were collected from patients attending the outpatient clinic at the Mona Vale Hospital (Mona Vale, NSW, n = 63), or solicited through media coverage and word-of-mouth (n = 397) from people experiencing tick-bite within Australia between 2013 and 2015. Information about the geographical location (Fig 1) and the date of the tick bite was obtained, and all ticks were confirmed (by medical history or questionnaire) to be actively blood feeding on humans at the time of removal. Ticks were preserved in 70% ethanol immediately after removal and morphologically identified into species, instar, and sex, at the Department of Medical Entomology, Westmead Hospital, or at Murdoch University, using standard keys [8, 17]. Tick specimens were then stored in 70% ethanol at 4°C until molecular analysis.

### DNA extraction

Total genomic DNA was extracted from individual ticks using the QIAGEN DNeasy Blood and Tissue Kit (QIAGEN, Germany) following the manufacturer’s recommendations (QIAGEN Supplementary Protocol: Purification of total DNA from insects). Before DNA extraction, the external surface of ticks was decontaminated in 10% hypochlorite solution, washed in sterile and DNA-free PBS, and 70% ethanol, and air-dried. Ticks were then frozen in liquid nitrogen for 1 minute, and homogenised by shaking with a 5 mm steel bead at 40 Hz for 1 minute. Extraction reagent blanks (EXB) (n = 20) were performed in parallel with all DNA extractions in order to establish background bacterial populations. All DNA extractions were performed in a physical isolation hood to minimise contamination by researchers and the environment, and sterile and DNA-free equipment was used for all procedures.

### Bacterial 16S rRNA gene profiling

The V1-2 hyper-variable region (250–320 bp) of bacterial 16S rRNA genes in tick DNA samples were PCR amplified using the primers 27F-Y and 338R as previously described [7]. These PCR assays for *I*. *holocyclus* DNA samples also included 10 μM of a “*Ca*. Midichloria mitochondrii”-specific blocking primer [7], in order to inhibit the amplification of 16S sequences from this highly abundant endosymbiotic bacterium. No-template (NT) and EXB controls were included in all PCR runs.

Amplicon library preparation was performed according to recommended protocols (Illumina Demonstrated Protocol: 16S Metagenomic Sequencing Library Preparation) with exceptions. Individual uniquely indexed libraries were normalised to equimolar concentrations with AxyPrep Mag PCR Normaliser beads (Axygen, USA) following the manufacturer’s recommendations, before pooling in equimolar amounts. Up to 96 uniquely indexed libraries were pooled per sequencing run, which were performed on an Illumina MiSeq using 500-cycle V2 chemistry (250 bp paired-end reads) following the manufacturer’s recommendations. No-template and EXB controls were also sequenced to establish background bacterial populations. All pre-PCR and post-PCR procedures were performed in physically separated laboratories to minimise amplicon contamination.

### Next Generation Sequencing Analysis

Sequences were first subjected to quality control procedures as previously described [7], with exceptions. Paired-end reads were merged using USEARCH v8.0.1623 [18] with a minimum overlap length of 50 bp and no gaps allowed in the merged alignments. Primer sequences and distal bases were trimmed from the ends of reads in Geneious v8.1.6 (Biomatters, New Zealand) [19] and reads shorter than the minimum previously reported length of the bacterial 16S V1-2 amplicon (< 250 bp) were removed. Singleton sequences (per sample) and sequences with a > 1% error rate were removed from the dataset using USEARCH v8.0.1623 [18]. Operational taxonomic units (OTUs) were created by clustering sequences at 97% similarity with the UPARSE algorithm [20], and taxonomy was assigned to OTUs in QIIME [21] by aligning to the GreenGenes 16S database (August 2013 release) [22] using the UCLUST algorithm [18] with default parameters. OTUs taxonomically assigned to the family or genus-level were used for further analysis. OTUs that were present in EXB and NT controls were removed from all samples in order to eliminate potentially contaminating and background bacteria.

Following OTU analysis to assign genus level taxonomy to 16S sequences, BLAST was used to resolve the species identity of families and genera that have medical or veterinary significance, or contain members that are known, or proposed, arthropod endosymbionts or pathogens. Species-level taxonomy was only inferred when the query matched 16S sequences from only one species with a ≥ 99% pairwise identity over ≥ 99% the length of the query sequence. Bacterial genera that were deemed not of medical or veterinary significance, known or proposed arthropod endosymbionts, or otherwise previously associated with ticks, and that were detected in less than the mean prevalence of all taxa, are herein not mentioned.

### Anaplasmataceae, *Borrelia*, and *Rickettsia*-specific PCR and Sanger sequencing

In order to gain more informative phylogenetic data and to verify NGS results, species-specific PCRs were used to further confirm (or refute) the occurrence of: *Borrelia* spp., Anaplasmataceae species (except *Wolbachia* spp.), and spotted fever and typhus group *Rickettsia* species in ticks. The *Borrelia*-specific assay targeted a 441 bp region of the chromosomal flagellin gene (*fla*B) and consisted of two nested PCRs, the primary reaction with primers *fla*B-280F and *fla*B-RL, and the nested reaction with primers *fla*B-LL and *fla*B-737R [23, 24], and verified previously in our laboratory to reliably amplify *B*. *burgdorferi* sensu lato, and relapsing fever group *Borrelia* spp. from tick specimens. The presence of Anaplasmataceae species in ticks was confirmed using a nested PCR assay targeting a 1.3 kb region of the 16S rRNA gene of Anaplasmataceae species (except *Wolbachia* spp.). The primary PCR contained the primers EC9 and EC12A [25, 26] and the nested reaction contained primers A17a and IS58-1345R [27]. The presence of spotted fever and typhus group *Rickettsia* species was confirmed with a qPCR assay using the primers CS-F and CS-R, and hydrolysis probe CS-P, as previously described [28].

*Borrelia* and Anaplasmataceae-specific primary PCRs contained 2 μl of tick DNA and the nested reaction used 1 μl of the primary PCR product as a template. PCRs contained PCR buffer, 2.5 mM MgCl_2_, 1 mM dNTPs, 0.01 mg BSA (Fisher Biotech, Australia), 1.25 U Perfect *Taq* Polymerase (5 Prime, Germany), and 400 nM of each primer, in a total volume of 25 μl. All PCRs included NT controls and positive controls (*B*. *afzelii* or “*Ca*. N. mikurensis” from *I*. *ricinus* ticks, and *R*. *australis* from culture). All positive PCR products were electrophoresed in 2% agarose gels stained with GelRed (Biotium, USA), visualised under UV light, purified with the QIAquick gel extraction kit (QIAGEN, Germany), and sequenced with both forward and reverse PCR primers on an ABI 3730 96 Capillary Sequences using Big dye v3.1 terminators (Life Technologies, USA).

### Anaplasmataceae 16S phylogenetic analysis

Phylogenetic analysis was conducted on 1,265bp 16S sequences obtained from the Anaplasmataceae-specific nested PCR on *I*. *holocyclus* and *A*. *triguttatum* samples, and additional Anaplasmataceae 16S sequences retrieved from GenBank. Sequences were aligned with MAFFT [29] and the gapped alignment was refined with MUSCLE [30]. The most suitable nucleotide substitution model was assessed in MEGA6 [31] and selected based on the Bayesian Information Criterion. Bayesian phylogenetic analysis was performed with the MrBayes software [32] using the HKY85 substitution model and a discrete Gamma distribution with 5 categories, a total chain length of 1,100,000, burn-in length of 100,000, and subsampling every 200 iterations.

## Results

### Bacterial 16S rRNA gene community profiling

The tick species collected from people while attached and feeding included *I*. *holocyclus* (*n* = 279), *A*. *triguttatum* (*n* = 167), *Haemaphysalis bancrofti* (*n* = 7), and *H*. *longicornis* (*n* = 7) (Table 1). *Ixodes holocyclus* ticks were received from almost the entirety of its enzootic range along the east coast of Australia from Gladstone, Queensland (QLD) to Mallacoota, Victoria (Fig 1). *Amblyomma triguttatum* ticks were primarily collected from southwest Western Australia (WA), including many semi-rural and rural areas surrounding Perth, as far north as Kalbarri, WA, and southeast at Hopetoun, WA (Fig 1). *Amblyomma triguttatum* ticks were also received from Rockhampton and Charleville, QLD (Fig 1). *Haemaphysalis longicornis* were collected from only a single location; Urunga, NSW, and *H*. *bancrofti* was collected from four locations, Gladstone, QLD, Currumbin, QLD, Mollymook, NSW, and Tamban, NSW (Fig 1).

**Table 1:** Summary of sample size, NGS coverage, and taxonomic diversity of tick species and life stages.

Tick Species	Instar/Sex	Number of samples	Total number of sequences	Mean sequences per sample	Number of bacterial genera^a^
*I*. *holocyclus*	Females	167	16,196,861	96,987.2	27
	Male	49	4,427,177	90,350.5	19
	Nymphs	63	3,188,402	50,609.5	22
*A*. *triguttatum*	Female	40	1,787,788	44,694.7	19
	Male	24	538,571	22,440.4	16
	Nymph	103	3,032,534	29,442.1	18
*H*. *bancrofti*	Male	1	13,284	13,284.0	16
	Nymph	6	145,742	24,290.3	15
*H*. *longicornis*	Female	3	72,635	24211.7	20
	Nymph	4	101,927	25,481.7	18
NT and EXB Controls		25	945,238	37,809.5	41

^a^ For tick samples only genera that were found in more than the mean prevalence of all taxa are shown

After NGS quality control procedures, 30,450,159 16S sequences from 460 tick samples and 25 NT and EXB control samples were used for analysis (Table 1). A total of 41 bacterial genera that were found in NT and EXB controls were removed from the dataset as background bacteria. All of the background taxa were either ubiquitous environmental or human-associated commensal bacterial genera that to the best of our knowledge have never been associated with tick-borne human or veterinary disease.

**Fig 2:**
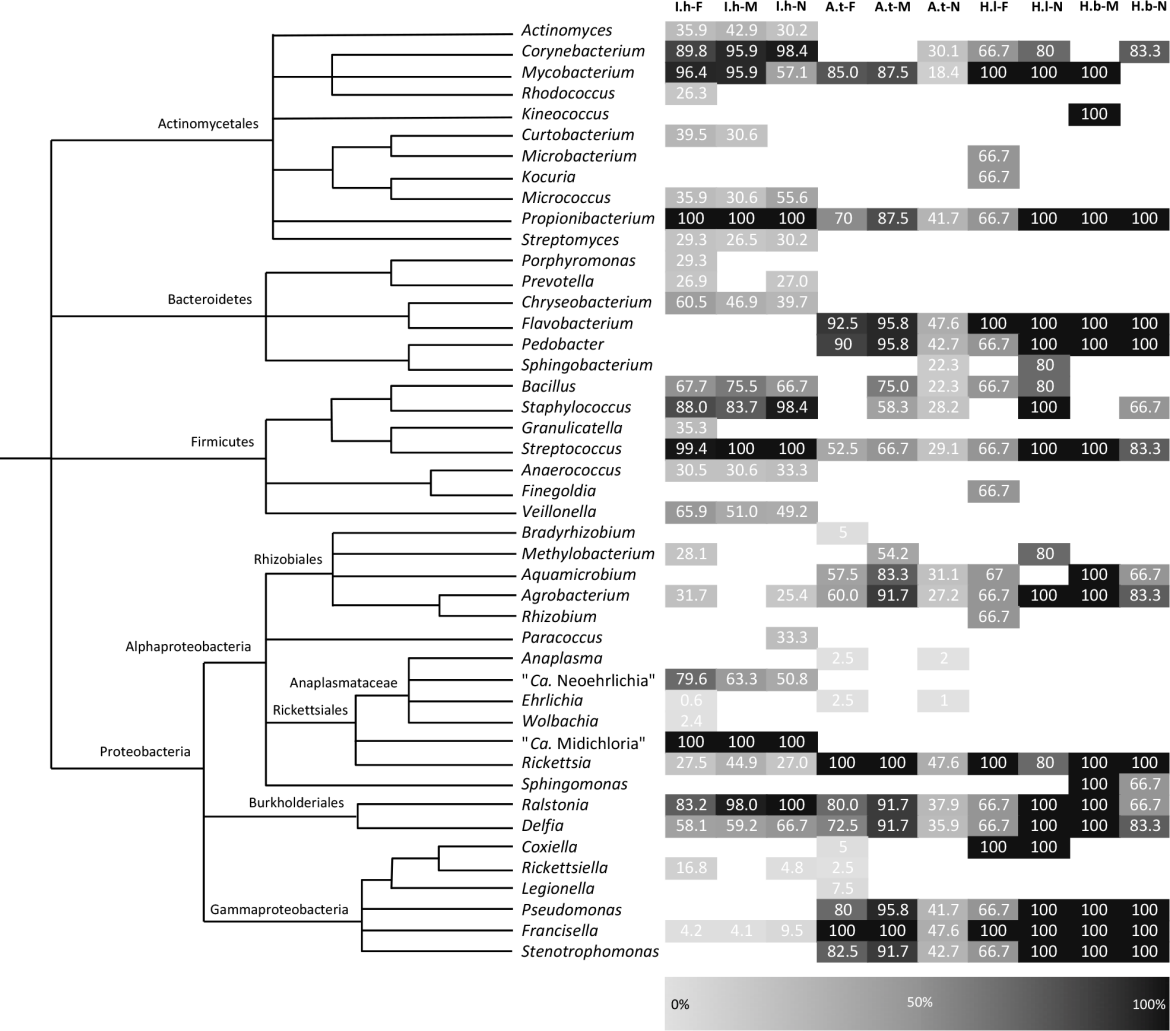
Cladogram and heat map showing the prevalence of bacterial genera in tick species and life stages. I.h, A.t, H.l, and H.b indicate *I*. *holocyclus*, *A*. *triguttatum*, *H*. *longicornis*, and *H*. *bancrofti* tick species, respectively. Female, Male and Nymph life stages are indicated by F, M, and N, respectively. The level of shading corresponds to the prevalence of the genera in the tick species and life stage. Blank shading indicates that bacterial genera were not detected in that tick species life stage.

The most prevalent organisms identified in *I*. *holocyclus*, *A*. *triguttatum*, *H*. *bancrofti*, and *H*. *longicornis* ticks were environmental or commensal bacteria that included 34 genera within Actinomycetales, Bacteriodetes, Firmicutes, Rhizobiales, Burkholderiales, and Gammaproteobacteria. The genera *Propionibacterium*, *Staphylococcus*, and *Streptococcus*, which live as commensals on mammalian skin were identified in all tick species (Fig 2). Other environmental genera identified, such as *Bacillus*, *Agrobacterium*, *Corynebacterium*, *Delftia*, *Flavobacterium*, *Methylobacterium*, *Mycobacterium*, *Pseudomonas*, *Ralstonia*, and *Stenotrophomones* are considered as either ubiquitous in the environment, or associated with soil and moist leaf litter environments in which ticks spend a large proportion of their life cycle (Fig 2). No *Borrelia* sp. sequences were identified in any of the 460 ticks.

#### Bacterial endosymbionts in human-biting ticks

Proposed bacterial endosymbionts were highly prevalent in all ticks studied, with each tick species having one or two predominant endosymbiont species and one to three less prevalent endosymbiotic associations. As anticipated from a previous study [7], the *Ixodes* tick endosymbiont “*Ca*. Midichloria mitochondrii” (16,519 unique sequences) was found in all *I*. *holocyclus* ticks, however, as expected (due to the use of a blocking primer during PCR) [7], 16S sequences from this abundant bacterium only comprised 4–17% of sequences per sample. In addition, *Wolbachia*, *Francisella*, and *Rickettsiella* spp. were also identified in 1.4%, 5.4%, and 11.1% of *I*. *holocyclus* ticks, respectively (Fig 2). Bacteria of the genus *Rickettsia* (7,069 unique sequences) were also identified in 27.5% of females, 44.9% of males, and 27% of nymph *I*. *holocyclus* ticks, with a total prevalence 30.5% in this tick species. Unfortunately *Rickettsia* 16S reads were unable to be given species designation due to high sequence homology (> 99%) between many *Rickettsia* species at the 16S locus analysed.

All *A*. *triguttatum*, *H*. *bancrofti*, and *H*. *longicornis* ticks studied were dual-infected with *Francisella* (12,990 unique sequences) and *Rickettsia* spp. (7,069 unique sequences) (Fig 2). *Francisella* and *Rickettsia* spp. sequences from these ticks were highly abundant in the NGS results, comprised between 12%-98% and 2%-88% of sequences per sample, respectively. *Francisella* sequences from all ticks were more than 98% similar to known endosymbiotic *Francisella* spp. from *A*. *maculatum* (GenBank: AY375407) and *Dermacentor* spp. (GenBank: AY375403, AY375401, JX101605) ticks from the northern hemisphere, and less than 94% similar to the infectious human pathogen *Francisella tularensis* (GenBank: NR074666), which has never been reported in Australia. In addition to endosymbiotic *Francisella* and *Rickettsia* spp., all *H*. *bancrofti* ticks also harboured a *Coxiella* sp., presumed to be an endosymbiont, as did 5% of *A*. *triguttatum* females. *Coxiella* sp. sequences were highly abundant in *H*. *bancrofti* ticks, comprising 23%-92% of sequences per sample. These *Coxiella* sp. sequences were more than 99% similar to *Coxiella* sp. endosymbionts reported previously from *H*. *lagrangei* and *H*. *longicornis* from Thailand and Korea (GenBank: KC170756, AY342036), respectively, but less than 94% similar to the infectious pathogen *C*. *burnetii* (GenBank: HG825990).

#### Novel Anaplasmataceae species identified in human-biting ticks

The genus “*Ca*. Neoehrlichia” (11,493 unique sequences) was identified in all *I*. *holocyclus* life stages studied, with a prevalence of 76.6%, 63.3%, and 50.8% in females, males, and nymphs, respectively, and a total prevalence of 88.9%. “*Candidatus* Neoehrlichia” sequences formed two distinct clusters, herein putatively named species A and B, which were 6–7% dissimilar from each other (S1 Table). The closest known relative to putative “*Ca*. Neoehrlichia” species A and B was “*Ca*. N. mikurensis” (94.6–94.9% similarity) (GenBank: AB196304) from Japan. Putative species A and B sequence were also highly similar to “*Ca*. N. lotoris” (95.9–96.3% similarity) (GenBank: EF633744), although the sequence query coverage was only 90.8%. Putative “*Ca*. Neoehrlichia” species A was most common, being found in 68.8% of “*Ca*. Neoehrlichia”-positive *I*. *holocyclus* ticks, compared to species B (31.2%). All sequences from both “*Ca*. Neoehrlichia” putative species A and B were more than 99% similar to “*Ca*. Neoehrlichia” spp. 16S sequences recently obtained by NGS from *I*. *holocyclus* ticks from NSW, Australia [7], with species A and B most similar to “*Ca*. Neoehrlichia” sp. isolates PI808 (GenBank: KT203915), and PI800 (GenBank: KT203914), respectively. Among all of the “*Ca*. Neoehrlichia”-positive *I*. *holocyclus* ticks, there were no cases of co-infection with both putative species A and B.

Interestingly, “*Ca*. Neoehrlichia” sequences were not detected in any *A*. *triguttatum* or *Haemaphysalis* ticks; however, two other Anaplasmataceae species were identified in *A*. *triguttatum* ticks and a single *I*. *holocyclus* female. Novel *Anaplasma* sp. sequences (284 unique sequences) were identified in three *A*. *triguttatum* ticks (1.8%), including one female (2.5%), and two nymphs (2%). These *Anaplasma* sp. sequences were most similar (98%) to an uncultured *Anaplasma* sp. (GenBank: JN862824) from southeast China, and the closest recognised species (97%) was *A*. *bovis* (GenBank: KJ659040). All three *A*. *triguttatum* ticks infected with this novel *Anaplasma* sp. originated from Yanchep National Park, Western Australia. Novel *Ehrlichia* sp. sequences (206 unique sequences) were also identified in two (1.2%) *A*. *triguttatum* ticks including one nymph, one female, and one *I*. *holocyclus* female (0.6%). These novel *Ehrlichia* sp. sequences were most similar (97%) to *E*. *ruminantium* (GenBank: DQ482921, CR925677), and another unresolved *Ehrlichia* sp. from *H*. *longicornis* ticks from Japan (GenBank: AY309970, HQ697588). The two *A*. *triguttatum* ticks infected with this novel *Ehrlichia* sp. both originated from Bullsbrook, Western Australia and the *I*. *holocyclus* tick originated from Pimpama, Queensland.

### Anaplasmataceae, *Borrelia*, and *Rickettsia*-specific PCR

All 460 *I*. *holocyclus*, *A*. *triguttatum*, and *Haemaphysalis* ticks were negative for *Borrelia* spp. by nested PCR, confirming the 16S community profiling results. The spotted fever and typhus group-specific qPCR did not amplify any *Rickettsia* from *I*. *holocyclus* ticks. However, all *Rickettsia*-positive *A*. *triguttatum* and *Haemaphysalis* ticks (by NGS) were amplified with this qPCR assay, indicating the *Rickettsia* spp. in these ticks are within, or closely related to spotted fever and typhus group *Rickettsia* species. The Anaplasmataceae-specific PCR assay returned 37 positive *I*. *holocyclus* ticks (12.9%), including 19 females (11.4%), eight males (16.3%), and 10 nymphs (15.9%), and five positive *A*. *triguttatum* ticks (3%), including two females (5%), and three nymphs (2.9%). No *Haemaphysalis* ticks were positive for Anaplasmataceae species.

### Anaplasmataceae Phylogenetic Analysis

**Fig 3:**
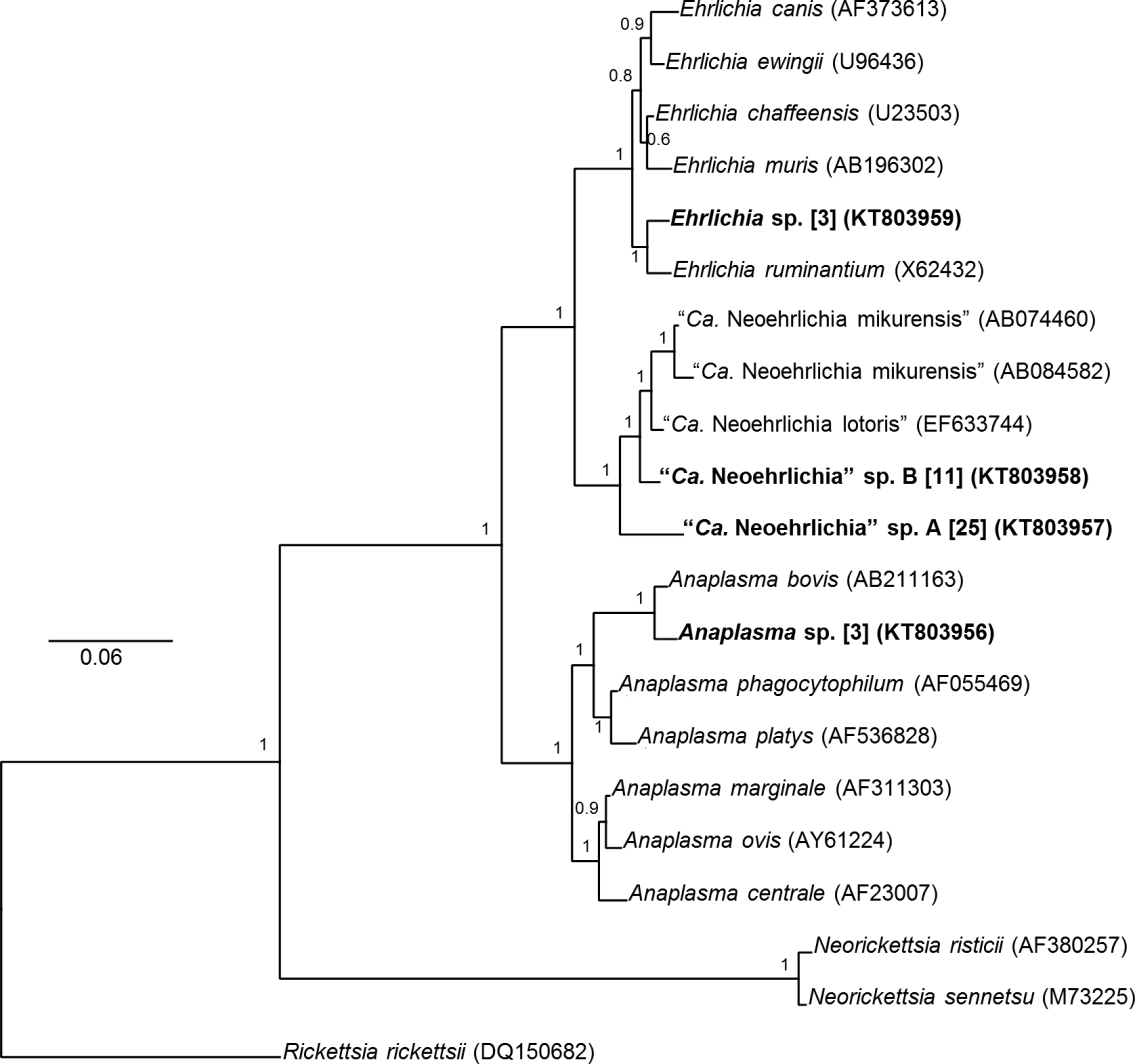
Bayesian phylogenetic analysis of 1,265 bp novel Anaplasmataceae 16S rRNA sequences from *I*. *holocyclus* and *A*. *triguttatum*. Bayesian posterior probabilities are displayed at each node. Bold type indicates sequences from this study. Rounded parentheses indicate GenBank accession numbers, and square parentheses indicate the number of ticks from which identical sequences were obtained.

Bayesian phylogenetic reconstruction of 1,265 bp 16S Anaplasmataceae sequences revealed that 36 (12.9% of all *I*. *holocyclus*) of the 37 positive *I*. *holocyclus* samples grouped with high confidence within the genus “*Ca*. Neoehrlichia”. Furthermore, the 16S sequences from these ticks clustered into two distinct groups, one containing identical sequences from 25 *I*. *holocyclus* ticks (9%) comprising putative “*Ca*. Neoehrlichia” species A (GenBank: KT803957), and the other containing identical sequences from 11 *I*. *holocyclus* ticks (4%), comprising putative “*Ca*. Neoehrlichia” species B (GenBank: KT803958) (Fig 3). Sequence from putative “*Ca*. Neoehrlichia” species A and B shared 96.2% similarity (S2 Table). The two known members of the genus, “*Ca*. N. lotoris” (GenBank: EF633744) and “*Ca*. N. mikurensis” (GenBank: AB074460, AB084582), were 98.1–98.6% similar at the 16S loci, however, putative “*Ca*. Neoehrlichia” species A and B were only 95.7–96.2%, and 97.3–98.4% similar to these species, respectively (S2 Table).

The level of divergence at the 16S loci both between putative “*Ca*. Neoehrlichia” species A and B, and between these and known “*Ca*. *Neoehrlichia*” spp., confirms the clustering pattern observed in the NGS data, and described previously [7]. All *I*. *holocyclus* ticks positive here for novel “*Ca*. Neoehrlichia” spp. were also positive for “*Ca*. Neoehrlichia” spp. by NGS, although the prevalence of “*Ca*. Neoehrlichia” spp. was significantly lower as determined by nested PCR (12.9%) than by NGS (88.9%).

Three identical novel *Anaplasma* sp. 16S sequences (GenBank: KT803956) from *A*. *triguttatum* ticks (1.8%), including one female and two nymphs, clustered with high confidence, but were distinct (98.7% similarity) from *A*. *bovis* (GenBank: AB211163) (Fig 3, S2 Table). In addition, a further three identical novel *Ehrlichia* sp. sequences (GenBank: KT803959) from two *A*. *triguttatum* ticks (1.2%), including one female (2.5%) and one nymph (0.97%), and one *I*. *holocyclus* female (0.6%) clustered with high confidence, but was distinct (98.3% similarity) from *E*. *ruminantium* (GenBank: X62432) (Fig 3, S2 Table). The level of divergence between these novel *Anaplasma* sp. and *Ehrlichia* sp. 16S sequences, and their closest relatives, is within the range of divergence among all *Anaplasma* species (94.7–99.4%) and *Ehrlichia* species (97.3–98.9%) (S2 Table). All ticks positive by nested PCR for novel *Anaplasma* sp. and *Ehrlichia* sp. were also positive for these taxa through NGS.

## Discussion

This study follows a preliminary investigation of the bacterial microbiome associated with *I*. *holocyclus* in a localised region of NSW, with the aim of investigating a collection of human-biting ticks over a greater geographical range, including areas of Sydney, NSW, where numerous patients have been diagnosed with a Lyme disease-like illness. In Australia, approximately eight species of hard ticks, and one species of soft tick (*Ornithodorus capensis*) are known to bite humans [8, 17, 33]. Consistent with previously published and anecdotal reports, the Australian paralysis tick (*I*. *holocyclus*) and the ornate kangaroo tick (*A*. *triguttatum*) were most frequently associated with attachment and engorgement on the skin of people in this study [8]. The introduced ‘bush’ tick, *H*. *longicornis*, normally a parasite of cattle, and the native wallaby tick (*H*. *bancrofti*) are also well known to bite people in Australia [8]. Curiously, we did not receive any specimens of the brown dog tick (*R*. *sanguineus*), the common marsupial tick (*I*. *tasmani*) or the southern paralysis tick (*I*. *cornuatus*) for analysis in this study, all of which have previously been associated with human tick bites in Australia.

Although the external cuticle of all ticks was decontaminated with ethanol and 10% hypochlorite solution prior to molecular analyses, a range of common environmental and commensal bacteria were still prevalent among all ticks surveyed. This is most likely due to remnant bacterial DNA that survived the decontamination process, perhaps in bacterial plaques that may have accumulated in less accessible places such as between leg joints or underneath the tick’s palps. In future studies careful dissection of the tick’s main internal tissues (midgut, salivary gland, and gonads) may prove useful in distinguishing the microbiome of the internal tissues from environmental bacteria on the tick’s external surfaces. Because all ticks surveyed were collected while actively feeding on humans it must be acknowledged that some bacteria in tick samples, such as *Staphylococcus* spp. and *Propionibacterium* spp., may have been from the blood and skin of the human hosts. However, most bacteria identified in the present study have been associated previously with ticks as members of genera that contain either known tick-borne pathogens, or arthropod endosymbionts.

Consistent with previous analysis [7], endosymbiotic “*Ca*. M. mitochondrii”, *Wolbachia*, *Francisella*, and *Rickettsia* spp. were identified in *I*. *holocyclus* ticks. All *A*. *triguttatum*, *H*. *bancrofti*, and *H*. *longicornis* ticks studied were dual-infected with endosymbiotic *Francisella* and *Rickettsia* spp., which comprised a large proportion of NGS sequencing output for these samples. Although *Francisella* endosymbionts have been described from northern hemisphere *Amblyomma* and *Dermacentor* ticks [34–36], and previously in *I*. *holocyclus* [7], this is the first description of *Francisella* spp. in a native Australian *Amblyomma* or *Haemaphysalis* tick. Species-specific blocking primers have been shown to be effective at inhibiting specific endosymbiont 16S sequences in *I*. *holocyclus* and *I*. *ricinus* [7], allowing the detection of less abundant bacterial taxa. It is probable that the use of *Francisella* and *Rickettsia*-specific blocking primers during 16S bacterial profiling of *A*. *triguttatum* and *Haemaphysalis* spp. ticks may similarly reveal more information about the less abundant bacterial taxa associated with these ticks.

The very high prevalence of *Rickettsia* spp. in *A*. *triguttatum* and *Haemaphysalis* ticks in this study suggest these *Rickettsia* spp. are likely endosymbiotic, and either advantageous or benign to the fitness of these tick species. The fact that these species were amplified with a qPCR assay designed to amplify only spotted fever and typhus group *Rickettsia* species and not the ancestral *R*. *bellii* species group [28], suggests these likely bacteria are more closely related to the spotted fever and typhus group than the *R*. *bellii* species group [37]. However, the spotted fever and typhus group qPCR did not amplify *Rickettsia* spp. found in *I*. *holocyclus* ticks, suggesting that these species are more closely related to the ancestral *R*. *bellii* group, which are typically endosymbionts of arthropods [37]. Further studies should include species-specific PCR and Sanger sequencing of a more informative marker gene to resolve the phylogenetic identity of *Rickettsia* spp. endosymbionts in Australian ticks, and to determine the prevalence of pathogenic *Rickettsia* spp. in Australia.

The absence of *Borrelia* sp. in the ticks studied here is somewhat unexpected considering the recent description of a single relapsing fever *Borrelia* sp. isolate found in a recent survey of *I*. *holocyclus* ticks using the same NGS method as in the present study. In that case the *Borrelia*-infected *I*. *holocyclus* tick was removed from an echidna, which is not a typical host for *I*. *holocyclus*. Surveying the microbial communities of ticks that share a close association with echidnas, such as *Bothriocroton concolor* and *B*. *hydrosauri*, may reveal more Australian *Borrelia* sp. isolates.

Based on the phylogenetic inference of 1,265 bp 16S sequences, the novel “*Ca*. Neoehrlichia”, *Ehrlichia*, and *Anaplasma* detected in the present study appear to be putative species, as the levels of divergence between their sequences and those of their closest relatives, is within the range of accepted species separation at the 16S rRNA gene loci [38–41]. However, formal descriptions of them as new species will require analysis at multiple loci such as the citrate synthase gene (gltA), RNA polymerase sub-unit β (*rpoB*) and heat shock operon (groESL), or whole genomes [27, 42–46].

The overall prevalence of novel “*Ca*. Neoehrlichia” species A and B across all *I*. *holocyclus* life stages was 88.9% by NGS but only 12.9% by nested PCR. There are several reasons that may explain this discrepancy; firstly the nested PCR amplified a large fragment (the primary amplicon was approximately 1.4 kb and the secondary amplicon was approximately 1.3 kb), which is known to reduce the efficiency of PCR [47]. For NGS, the amplicon size was much smaller (250–320 bp) and would therefore be expected to amplify with much greater efficiency [47, 48]. Secondly, NGS allows the detection of low abundant sequences, and mixed sequences that would not be detected with Sanger sequencing [48]. Further studies should include use a “*Ca*. Neoehrlichia”-specific droplet digital PCR quantitation assay targeting small amplicon sizes, as this will allow for more accurate quantitation [49, 50] and determination of the true prevalence of novel “*Ca*. Neoehrlichia” species in *I*. *holocyclus*.

All recognised members of the genera *Anaplasma*, *Ehrlichia*, and *“Ca*. Neoehrlichia” are obligate intracellular tick-borne mammalian pathogens that typically infect haematopoietic (mammalian) or endothelial (mammalian and tick) cells [25, 51–53]. There has been no confirmed transovarial transmission of *Anaplasma*, *Ehrlichia*, or “*Ca*. Neoehrlichia” species in vector-ticks or mammals, and therefore their persistence is attributed predominantly to infected mammalian reservoir populations [51–53]. Throughout Europe, Asia, and North America several Anaplasmataceae species are pathogens of veterinary significance (such as *E*. *canis* and *E*. *ruminantium*) and important emerging human pathogens, such as *E*. *chaffeensis*, *E*. *ewingii*, *A*. *phagocytophilum*, and “*Ca*. N. mikurensis”

“*Ca*. Neoehrlichia” is a recently described genus that currently comprises two species, “*Ca*. N. lotoris”, and “*Ca*. N. mikurensis” [27, 45]. Of these “*Ca*. N. mikurensis” is now recognised an emerging tick-borne zoonosis vectored by several tick species (*I*. *ricinus*, *I*. *ovatus*, and *I*. *persulcatus*), and is one of the most prevalent tick-borne infections in wildlife and ticks throughout Europe and Asia [27, 35, 54–68]. Clinical reports of human infections are steadily increasing, due in part to increased awareness and testing [53]. Infection with “*Ca*. N. mikurensis” (neoehrlichiosis) is typically severe, with a wide variety of non-specific symptoms reported [69–75]. In Europe, neoehrlichiosis usually manifests in immunocompromised patients, however in China, there are increasing reports of this infection in immunocompetent people, and asymptomatic infections in humans have also been reported [76, 77]. In contrast, “*Ca*. N. lotoris” is a tick-borne pathogen of racoons (*Procyon lotor*), and to date there are no reports of human infection [45, 78]. In the northern hemisphere treatment of patients suffering neoehrlichiosis with doxycycline (1 x 200 mg/day) for 3–6 weeks has been shown to be effective [71, 75, 79, 80], and may have implication if human or animals infections are found to occur in Australia.

The identification of four novel putative tick-borne Anaplasmataceae species in Australian human-biting ticks is of potential public health significance, especially the high prevalence of novel “*Ca*. Neoehrlichia” spp. in *I*. *holocyclus* ticks. Based on their phylogenetic position, as inferred here, and the disease-causing status of their close relatives, all four species are candidate human and animal pathogens, and almost certainly infective (symptomatic or asymptomatic) to Australian wildlife species. Determining whether these *Ehrlichia*, *Anaplasma* and “*Ca*. Neoehrlichia” species may cause disease in Australian humans, like their close relatives do overseas is of public health importance. Future studies should include the development of specific digital and qPCR assays to more accurately determine the prevalence and pathogen load in ticks, wildlife, and humans. In addition, the isolation and culture of these organisms, in pure culture or infected mammalian and tick cell culture, will significantly aid in understanding the biology and potential pathogenicity of these novel Anaplasmataceae, and the development of specific diagnostic serological test and therapeutic practices.

## Supporting Information

S1 Table Distance matrix of the pairwise percent similarity of the 10 most prevalent 16S V1-2 sequences from “*Ca*. Neoehrlichia” putative species A (A1-10) and B (B1-10). Shading indicated > 99% similarity between sequences in putative “*Ca*. Neoehrlichia” spp. A and B.
(PDF)

S2 Table Pairwise percentage distance matrix of 1,265 bp Anaplasmataceae 16S sequences from this study and retrieved from GenBank used for Bayesian phylogenetic reconstruction. (PDF)
