# Supplementary material for: Bacterial Profiling Reveals Novel “Ca. Neoehrlichia”, Ehrlichia, and Anaplasma Species in Australian Human-Biting Ticks
Source: PLoS One. 2015 Dec 28;10(12):e0145449. doi: 10.1371/journal.pone.0145449 (PMC4692421; doi:10.1371/journal.pone.0145449)
Supplement: S2 Table — (PDF) [file pone.0145449.s002.pdf]

S2 Table. Pairwise percentage distance matrix of 1,265 bp Anaplasmataceae 16S sequences from this study and retrieved from GenBank used for Bayesian phylogenetic reconstruction.

	A. bovis (AB211163)	A. centrale (AF23007)	A. marginale (AF311303)	A. ovis (AY261224)	A. phagocytophilum (AY055469)	A. platys (AF536828)	Anaplasma sp. (KT803956)	Ehrlichia sp. (KT803959)	"Ca. Neoehrlichia mikurensis" (AB074 460)	"Ca. Neoehrlichia" sp. A (KT803957)	"Ca. Neoehrlichia" sp. B (KT803958)	Ca. Neoehrlichia sp. B	"Ca. Neoehrlichia lotoris" (EF633744)	E. canis (AF373613)	E. chaffeensis (U23503)	E. ewingii (U96436)	E. muris (AB196302)	E. ruminantium (X62432)	Neorickettsia risticii (AF380257)	Neorickettsia sennetsu (M73225)	Rickettsia rickettsii (DQ150682)
A. bovis (AB211163)																					
A. centrale (AF23007)	95.1																				
A. marginale (AF311303)	95.8	98.7																			
A. ovis (AY261224)	95.7	98.3	99.4																		
A. phagocytophilum (AY055469)	96.6	96.5	97.2	96.9																	
A. platys (AF536828)	96.5	96.2	96.7	96.5	98.8																
Anaplasma sp. (KT803956)	98.7	94.7	95.6	95.5	96.3	96.2															
Ehrlichia sp. (KT803959)	91	91.9	92.5	92.1	92	91.5	91.4														
"Ca. Neoehrlichia mikurensis" (AB074460)	91.4	92.4	92.4	92.5	92.8	92.4	91.3	94.1													
"Ca. Neoehrlichia mikurensis" (AB084582)	91	91.9	91.9	92	92.6	91.9	91.1	93.8	99.2												
"Ca. Neoehrlichia" sp. A (KT803957)	90.7	92.3	92.3	92.4	91.8	91.2	90.8	94.4	96.2	95.7											
"Ca. Neoehrlichia" sp. B (KT803958)	91.5	92.7	92.7	92.4	92.8	92.4	91.5	94.8	97.8	97.3	96.2										
"Ca. Neoehrlichia lotoris" (EF633744)	91.5	92.8	92.7	92.6	93.1	92.4	91.3	94.2	98.6	98.1	96.2	98.4									
E. canis (AF373613)	90.8	92.2	92.6	92.3	92.2	91.5	90.9	97.4	93.4	93	93.4	94.4	93.8								
E. chaffeensis (U23503)	91.3	92.4	93	92.7	92.8	92.2	91.5	98	94.2	93.8	94.2	94.7	94.6	98.5							
E. ewingii (U96436)	90.8	92.1	92.6	92.1	92.5	91.8	91.1	98	94.1	94	94.1	94.7	94.4	98.5	98.9						
E. muris (AB196302)	91.2	92.3	92.7	92.4	92.3	92	91.2	97.7	94.4	94.1	94.4	94.8	94.4	97.7	98.9	98.4					
E. ruminantium (X62432)	91	92	92.3	92	92.4	92	90.9	98.3	94.5	94.3	94.3	94.9	94.7	97.3	98	98.2	97.6				
Neorickettsia risticii (AF380257)	84.3	84.8	85	84.7	85.3	84.9	84	83.9	84	83.6	83.2	84.1	84.5	84.3	84.7	84.4	84.3	84.5			
Neorickettsia sennetsu (M73225)	84.4	85	85.2	84.9	85.6	85.1	84.3	84.3	84.2	83.8	83.4	84.5	84.8	84.7	85.1	84.8	84.7	84.9	99.2		
Rickettsia rickettsii (DQ150682)	82.6	83.9	83.7	83.4	84	83.1	82.5	83.4	84.1	83.7	82.7	83.7	83.7	82.8	83	83.1	82.6	83.6	81.6	81.9	
