# Supplementary material for: Bacterial Profiling Reveals Novel “Ca. Neoehrlichia”, Ehrlichia, and Anaplasma Species in Australian Human-Biting Ticks
Source: PLoS One. 2015 Dec 28;10(12):e0145449. doi: 10.1371/journal.pone.0145449 (PMC4692421; doi:10.1371/journal.pone.0145449)
Supplement: S1 Table — Shading indicated > 99% similarity between sequences in putative “Ca. Neoehrlichia” spp. A and B. (PDF) [file pone.0145449.s001.pdf]

**S1 Table. Distance matrix of the pairwise precent similarity of the 10 most prevalent 16S V1-2 sequences from "*Ca.* Neoehrlichia" putative species A (A1-10) and B (B1-10).** Shading indicates > 99% similarity between sequences in putative "*Ca.* Neoehrlichia" spp. A and B.

| | A1 | A2 | A3 | A4 | A5 | A6 | A7 | A8 | A9 | A10 | B1 | B2 | B3 | B4 | B5 | B6 | B7 | B8 | B9 | B10 |
|---|---|---|---|---|---|---|---|---|---|---|---|---|---|---|---|---|---|---|---|---|
| A1 | | | | | | | | | | | | | | | | | | | | |
| A2 | 99.3 | | | | | | | | | | | | | | | | | | | |
| A3 | 99.3 | 99.3 | | | | | | | | | | | | | | | | | | |
| A4 | 99.3 | 99.3 | 99.3 | | | | | | | | | | | | | | | | | |
| A5 | 99.3 | 99.3 | 99.3 | 99.3 | | | | | | | | | | | | | | | | |
| A6 | 99.3 | 99.3 | 99.3 | 99.3 | 99.3 | | | | | | | | | | | | | | | |
| A7 | 99.3 | 99.3 | 99.3 | 99.3 | 99.3 | 99.3 | | | | | | | | | | | | | | |
| A8 | 99.3 | 99.3 | 99.3 | 99.3 | 99.3 | 99.3 | 99.3 | | | | | | | | | | | | | |
| A9 | 99.3 | 99.3 | 99.3 | 99.3 | 99.3 | 99.3 | 99.3 | 99.3 | | | | | | | | | | | | |
| A10 | 99.3 | 99.3 | 99.3 | 99.3 | 99.3 | 99.3 | 99.3 | 99.3 | 99.3 | | | | | | | | | | | |
| B1 | 92.9 | 92.9 | 92.9 | 92.9 | 93.6 | 92.9 | 92.9 | 92.9 | 92.9 | 92.9 | | | | | | | | | | |
| B2 | 92.9 | 92.9 | 92.9 | 92.9 | 93.6 | 92.9 | 92.9 | 92.9 | 92.9 | 92.9 | 99.7 | | | | | | | | | |
| B3 | 92.9 | 92.9 | 92.9 | 92.9 | 93.6 | 92.9 | 92.9 | 92.9 | 92.9 | 92.9 | 99.3 | 99.3 | | | | | | | | |
| B4 | 92.9 | 92.9 | 92.9 | 92.9 | 93.6 | 92.9 | 92.9 | 92.9 | 92.9 | 92.9 | 99.3 | 99.3 | 99.7 | | | | | | | |
| B5 | 92.9 | 92.9 | 92.9 | 92.9 | 93.6 | 92.9 | 92.9 | 92.9 | 92.9 | 92.9 | 99.3 | 99.3 | 99.3 | 99.3 | | | | | | |
| B6 | 92.9 | 92.9 | 92.9 | 92.9 | 93.6 | 92.9 | 92.9 | 92.9 | 92.9 | 92.9 | 99.3 | 99.3 | 99.3 | 99.3 | 99.7 | | | | | |
| B7 | 92.9 | 92.9 | 92.9 | 92.9 | 93.6 | 92.9 | 92.9 | 92.9 | 92.9 | 92.9 | 99.3 | 99.3 | 99.3 | 99.3 | 99.3 | 99.3 | | | | |
| B8 | 92.9 | 92.9 | 92.9 | 92.9 | 93.6 | 92.9 | 92.9 | 92.9 | 92.9 | 92.9 | 99.3 | 99.3 | 99.3 | 99.3 | 99.3 | 99.3 | 99.7 | | | |
| B9 | 92.9 | 92.9 | 92.9 | 92.9 | 93.6 | 92.9 | 92.9 | 92.9 | 92.9 | 92.9 | 99.3 | 99.3 | 99.3 | 99.3 | 99.3 | 99.3 | 99.3 | 99.3 | | |
| B10 | 92.9 | 92.9 | 92.9 | 92.9 | 93.6 | 92.9 | 92.9 | 92.9 | 92.9 | 92.9 | 99.3 | 99.3 | 99.3 | 99.3 | 99.3 | 99.3 | 99.3 | 99.3 | 99.7 | |
